# *Oberholzeria* (Fabaceae subfam. Faboideae), a New Monotypic Legume Genus from Namibia

**DOI:** 10.1371/journal.pone.0122080

**Published:** 2015-03-27

**Authors:** Wessel Swanepoel, M. Marianne le Roux, Martin F. Wojciechowski, Abraham E. van Wyk

**Affiliations:** 1 Independent Researcher, Windhoek, Namibia; 2 H. G. W. J. Schweickerdt Herbarium, Department of Plant Science, University of Pretoria, Pretoria, South Africa; 3 Department of Botany and Plant Biotechnology, University of Johannesburg, Johannesburg, South Africa; 4 School of Life Sciences, Arizona State University, Tempe, Arizona, United States of America; University of Delhi, INDIA

## Abstract

*Oberholzeria etendekaensis*, a succulent biennial or short-lived perennial shrublet is described as a new species, and a new monotypic genus. Discovered in 2012, it is a rare species known only from a single locality in the Kaokoveld Centre of Plant Endemism, north-western Namibia. Phylogenetic analyses of molecular sequence data from the plastid *mat*K gene resolves *Oberholzeria* as the sister group to the Genisteae clade while data from the nuclear rDNA ITS region showed that it is sister to a clade comprising both the Crotalarieae and Genisteae clades. Morphological characters diagnostic of the new genus include: 1) succulent stems with woody remains; 2) pinnately trifoliolate, fleshy leaves; 3) monadelphous stamens in a sheath that is fused above; 4) dimorphic anthers with five long, basifixed anthers alternating with five short, dorsifixed anthers, and 5) pendent, membranous, one-seeded, laterally flattened, slightly inflated but indehiscent fruits.

## Introduction

**Fig 1:**
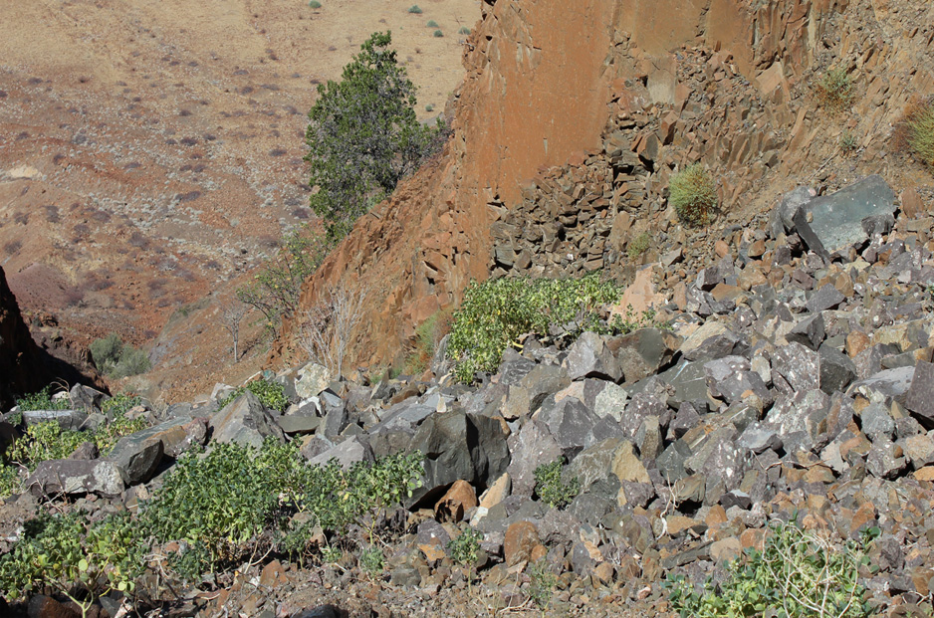
Natural habitat of *Oberholzeria etendekaensis*. Plants of *O*. *etendekaensis* in their natural habitat (low-growing shrublets in foreground), Etendeka Mountains, Namibia. The plants grow in stony soil and scree derived from basalt of the Etendeka Group, Karoo Supergroup. This section of the Great Escarpment lies to the east of the Namib Desert, about 50 km from the Atlantic Ocean coastline. The climate is very arid, with an average annual rainfall of about 100 mm. Photo: W. Swanepoel.

**Fig 2:**
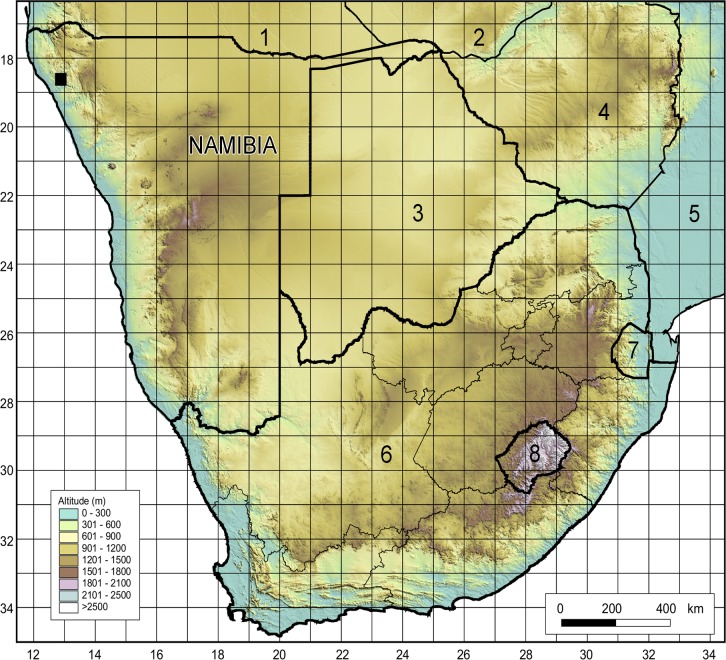
Geographical distribution of *Oberholzeria etendekaensis*. Topographic map of southern Africa showing the known distribution of *O*. *etendekaensis* (black square) in the far north-western corner of Namibia. The locality falls within the Kaokoveld Centre of Endemism, a biogeographical region rich in restricted-range plants and animals. The new species is known from a single population comprising two small subpopulations growing about 500 m apart in the Etendeka Mountains. These mountains form part of the Great Escarpment of southern Africa. Neighbouring countries indicated by numbers, namely Angola (1), Zambia (2), Botswana (3), Zimbabwe (4), Mozambique (5), South Africa (6), Swaziland (7) and Lesotho (8).

The Fabaceae subfam. Faboideae (Leguminosae subfam. Papilionoideae) is represented in Namibia by 52 genera and ca. 255 species [1], none of which are succulent. In May 2012, during a plant collecting expedition to the far north-western corner of Namibia, a region known as the Kaokoveld, the first author encountered an unusual papilionoid legume but which superficially resembles a member of *Zygophyllum* L. [2] (Zygophyllaceae). The plants were found in two small subpopulations in the Etendeka Mountains (Fig. 1) which form part of the Great Escarpment of southern Africa (Fig. 2).

The taxonomic placement of the new genus is uncertain as it shares morphological characters with both the Crotalarieae (calyces with five equal lobes, rostrate keels, dimorphic anthers with a 5 + 5 arrangement, and slightly inflated, pendent fruits) [3–8] and the Genisteae (lack of an aril, stamen filaments fused into a closed tube and strongly dimorphic anthers) [3, 4, 9–11], but differs from both tribes in being succulent.

Over the past several years, quite a few adjustments were made to the circumscription of these two tribes and several genera were transferred from one tribe to another, e.g. *Anarthrophylum* Benth. [12], *Argyrolobium* Eckl. & Zeyh. [13], *Dichilus* DC. [14] and *Melolobium* Eckl. & Zeyh. [13] were moved from the Crotalarieae to Genisteae [15]. These updated tribal circumscriptions have been confirmed by molecular studies [16–21] and show that both tribes form part of the Genistoid s.l. clade together with the Brongniartieae, Podalyrieae and the newly instated tribes Leptolobieae and Ormosieae [11].

In the present contribution the unusual papilionoid legume from Namibia is formally described as a new genus and species, namely *Oberholzeria etendekaensis*. Included are a diagnosis, morphological description, distribution map, line drawings and photographs, as well as two molecular phylogenies (nuclear rDNA ITS and plastid *mat*K) which aid in determining the phylogenetic placement of the new genus.

## Materials and Methods

### Ethics statement

The collection location for the new species reported in this work is not protected in any way. The species described here is currently not included in the Namibian Red Data Book. Material of the new species was collected under permit no. 1697/2012, issued to one of us (WS) by the Ministry of Environment and Tourism, Republic of Namibia.

### Nomenclature

The electronic version of this article in Portable Document Format (PDF) in a work with an ISSN or ISBN will represent a published work according to the International Code of Nomenclature for algae, fungi, and plants, and hence the new names contained in the electronic publication of a PLOS ONE article are effectively published under that Code from the electronic edition alone, so there is no longer any need to provide printed copies.

In addition, new names contained in this work have been submitted to IPNI, from where they will be made available to the Global Names Index. The IPNI LSIDs can be resolved and the associated information viewed through any standard web browser by appending the LSID contained in this publication to the prefix http://ipni.org/. The online version of this work is archived and available from the following digital repositories: PubMed Central, LOCKSS.

### Morphological observations

The morphological description of the new genus was based on examination of fresh specimens. Details of the flowers were examined under a stereomicroscope. The morphological comparison with other species of the subfam. Faboideae was based on the study of live plants in the field as well as in cultivation, herbarium specimens, and information gathered from the literature. Newly collected specimens have been deposited in the herbarium of the National Botanical Research Institute, Windhoek, Namibia (WIND) and the National Herbarium, Pretoria, South Africa (PRE).

### Taxon sampling and DNA sequencing

Genomic DNA was extracted from selected herbarium specimens and dried leaf material using DNeasy Plant Minikits (Qiagen, Valencia, California, USA). DNA samples for *Melolobium exudans* Harv. and *Polhillia obsoleta* (Harv.) B.-E. van Wyk were obtained from the DNA Bank at the Royal Botanic Gardens, Kew, UK. The nuclear rDNA ITS region and complete plastid *mat*K gene sequences were amplified by polymerase chain reaction methods as described previously [20, 22]. DNA sequencing was performed at the High Throughput Genomics Center (Seattle, Washington, USA). Sequence output files were assembled into contigs and edited using the program Sequencher 4.9 (GeneCodes, Ann Arbor, Michigan, USA) before alignment. For both loci, primers were used for sequencing reactions in both directions to generate complete overlap (100%) in the assembly of sequences. The sources of plant material used for all new sequences and GenBank information for sequences (both ITS and *mat*K) from all taxa included in this paper are provided in S1, S2 and S3 Tables respectively. The ITS and *mat*K datasets were submitted to TreeBase (submission number 16779, accessible at the URL http://purl.org/phylo/treebase/phylows/study/TB2:S1677).

Complete nrDNA ITS and *mat*K gene sequences were newly obtained from 11 taxa, including two collections of *Oberholzeria*, for this study. The new nrDNA ITS sequences were added to a data set (partially compiled from an existing dataset [23] and sequences retrieved from Genbank), then aligned manually and reduced to 109 taxa before analyses. The new *mat*K sequences were provisionally aligned with an updated version of *mat*K data set [20], which was then reduced in size to include only the representative papilionoids analysed here (74 taxa). Gaps in both data sets were treated as missing data and excluded from all analyses.

### Phylogenetic analysis

The nrDNA ITS and *mat*K data sets were analyzed separately, due to lack of significant taxonomic overlap. Maximum parsimony (MP) analyses were performed using PAUP* 4.0b10 [24]. Multiple tree searches were conducted using heuristic search options that included SIMPLE, CLOSEST, and RANDOM addition sequences (1000 replicates) holding 1–5 trees per replicate, and tree-bisection-reconnection (TBR) branch swapping, with retention of multiple parsimonious trees (MAXTREES = 1000 initially). Non-parametric bootstrap [25] proportions (BS) were estimated from 100–500 bootstrap replicates for each data set, incorporating heuristic search options as used in the standard parsimony searches.

Data sets were also analyzed by Bayesian inference [26] using a general time reversible model with gamma shape parameter and proportion of invariant sites (GTR + I + Γ) selected as the best model for both the nrDNA ITS and *mat*K data sets based on the Akaike Information Criterion (AIC) in MrModeltest (version 2) [27]. Bayesian analyses were run for 5 × 10^6^ generations with four chains, sampling every 5 × 10^3^ generations, using uniform (default) priors. Trees saved prior to stationarity were excluded by “burnin” (25% of samples) and the remaining 750 trees were used to construct a majority rule consensus tree with clade credibility values (posterior probabilities; PP).

## Results

### Phylogenetic analysis

#### ITS nuclear data

**Fig 3:**
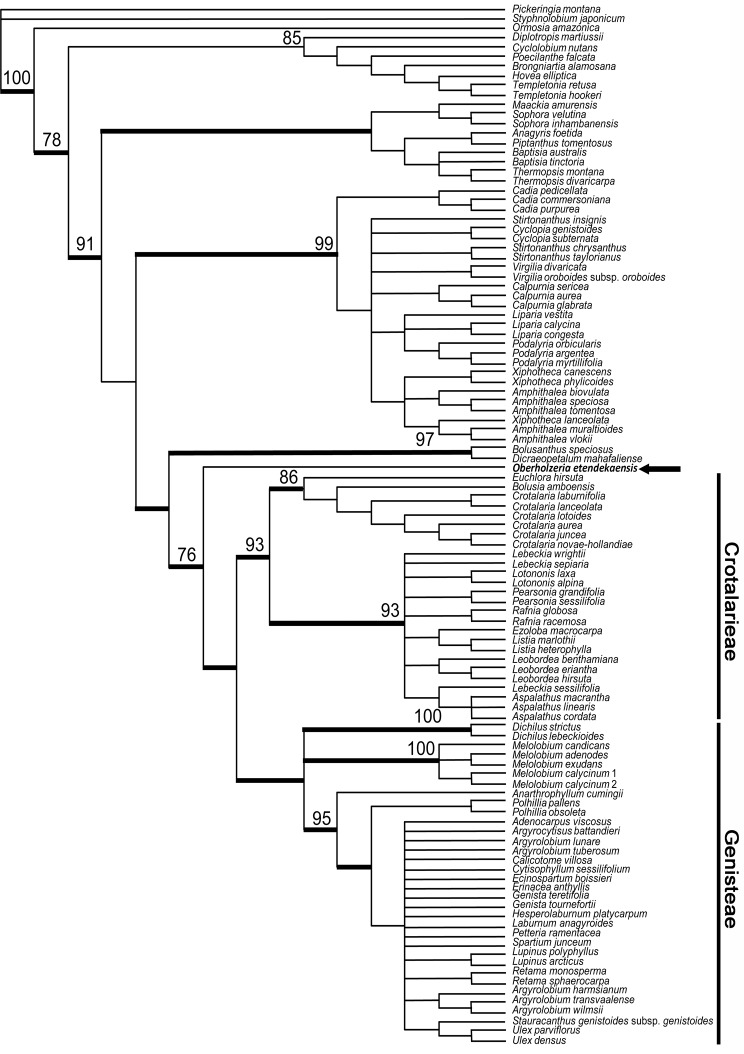
Phylogenetic tree based on nrDNA ITS sequences. Phylogenetic relationship of *Oberholzeria etendekaensis* (arrowed) derived from maximum parsimony analysis of the nrDNA ITS sequences; 109 taxa, 740 total characters with 639 included, of which 330 (52%) were parsimony informative. Tree shown is strict consensus of >100,000 equally most parsimonious trees of 1784 steps. Numbers represent maximum parsimony bootstrap support values (100–500 replicates) greater than 70% for selected clades; thickened branches represent clades with Bayesian posterior probabilities greater than 0.95. *Oberholzeria* is supported as the sister to a clade comprised of tribes Crotalarieae and Genisteae (76% and 1.0, respectively).

The ITS data set (Fig. 3) consisted of 109 taxa and 639 included positions and the MP analysis produced >100,000 trees of 1784 steps (CI = 0.4187, RI = 0.7243). Results of both bootstrap and Bayesian analyses show *Oberholzeria* is supported as the sister to a clade comprising tribes Crotalarieae and Genisteae (76% and 1.0, respectively).

#### *mat*K plastid data

**Fig 4:**
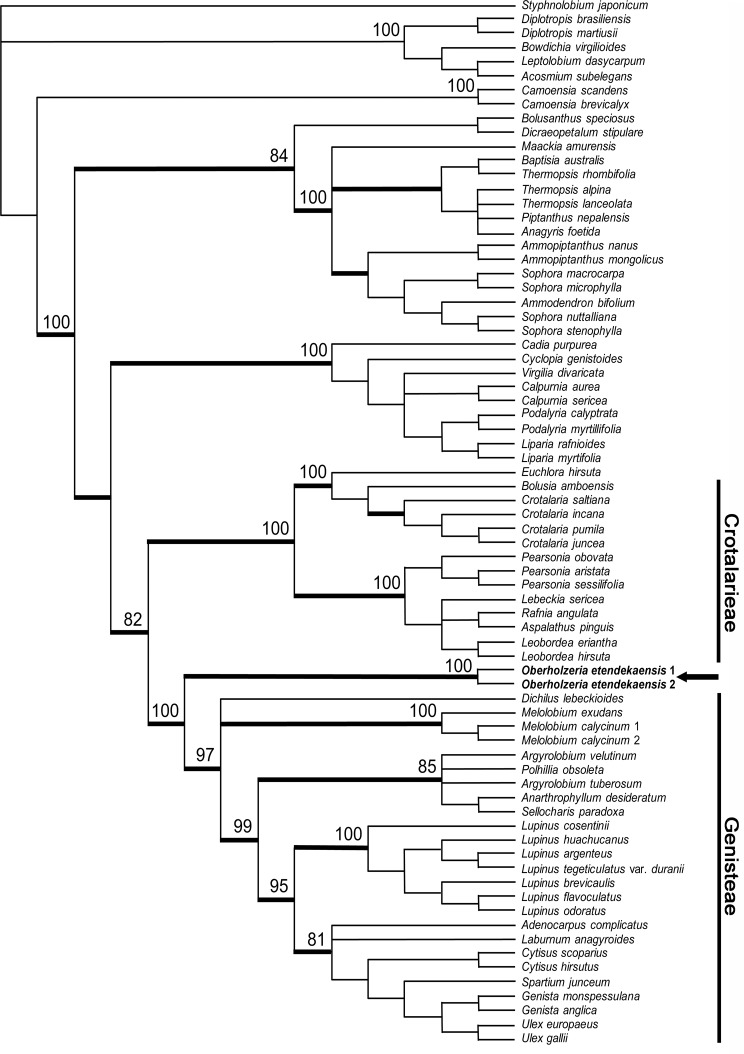
Phylogenetic tree based on plastid *mat*K gene sequences. Phylogenetic relationship of *Oberholzeria etendekaensis* (arrowed) derived from maximum parsimony analysis of the plastid *mat*K gene sequences; 74 taxa, 1572 total characters with 1518 included, of which 456 (30%) were parsimony informative. Tree shown is strict consensus of 794 equally most parsimonious trees of 1076 steps. Numbers represent maximum parsimony bootstrap support values (500 replicates) greater than 70% for selected clades; thickened branches represent clades with Bayesian posterior probabilities greater than 0.95. Both bootstrap and Bayesian analyses strongly support *Oberholzeria* as the sister group to the Genisteae clade (100% and 1.0, respectively).

Maximum parsimony analysis was conducted on the *mat*K data set (Fig. 4), which consisted of 74 taxa and 1518 included positions, and produced 794 trees of 1076 steps (CI = 0.6988, RI = 0.8885); Table 1. Both bootstrap and Bayesian analyses strongly support *Oberholzeria* as the sister group to the Genisteae clade (100% and 1.0, respectively).

**Table 1:** Summary of the statistics of the phylogenetic analyses that were conducted on both the ITS and *mat*K datasets.

	Number of taxa	Number of aligned characters	Number of included characters	Number of parsimony informative characters	Number of steps	Number of trees	CI	RI
**ITS**	109	740	639	330 (52%)	1784	>100,000	0.4187	0.7243
***mat*K**	74	1572	1518	456 (30%)	1076	794	0.6988	0.8885

## Discussion

**Fig 5:**
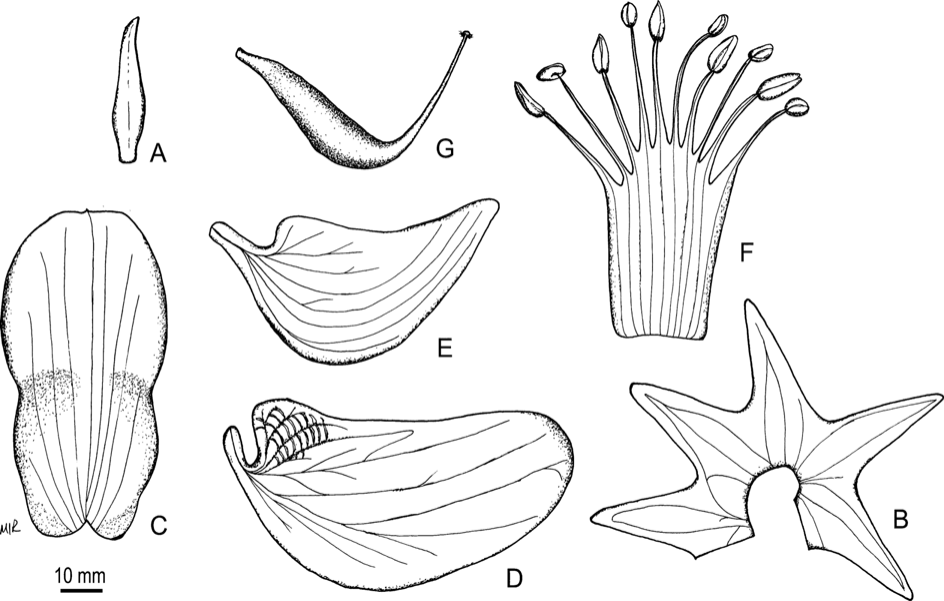
Flower morphology of *Oberholzeria etendekaensis*. Line drawings depicting the flower morphology. (A) Bract. (B) Calyx, opened out; lobes equal, dorsiventrally flattened. (C) Standard; strongly reflexed in the intact flower and lacking callosities. (D) Wing petal; longer than the keel and without a spur. (E) Keel petal. (F) Androecium, opened out; diagnostic for the genus is the stamens which are all fused into a tube that is closed above, and dimorphic anthers with five long, basifixed anthers alternating with five short, dorsifixed anthers. (G) Gynoecium. Voucher: *Swanepoel 316* (WIND). Artist: M.M. le Roux.

**Fig 6:**
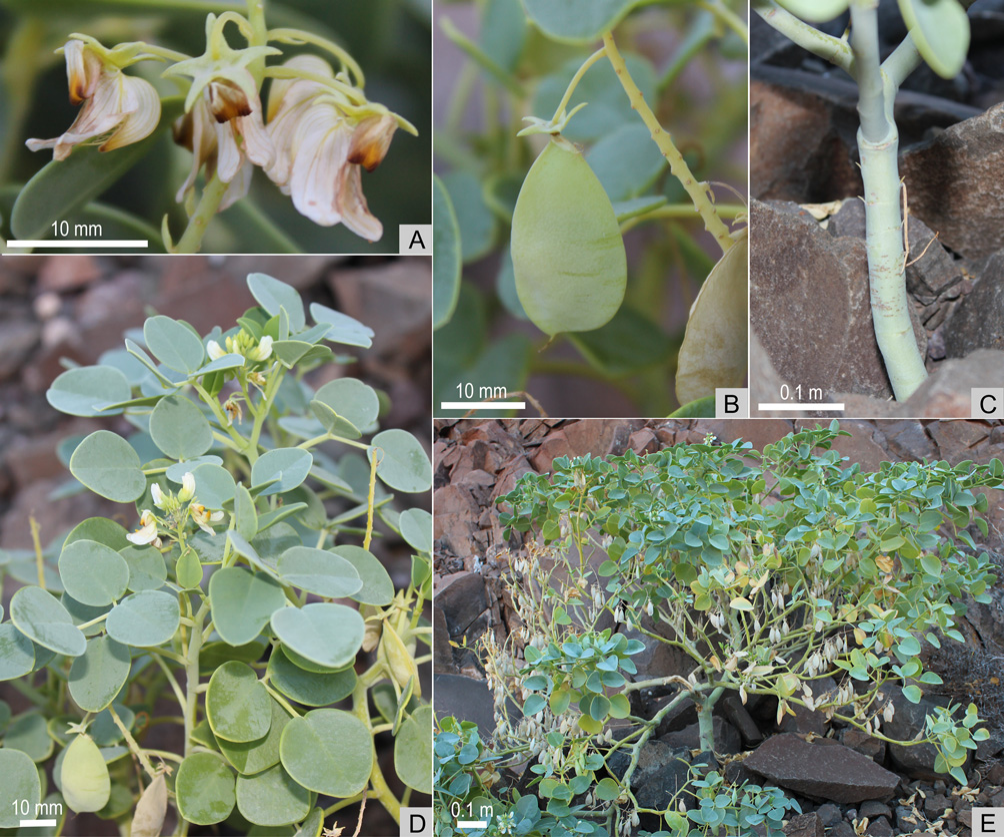
Habit and macromorphology of *Oberholzeria etendekaensis*. Photographs illustrating the morphology of the plants. (A) Flowers with rostrate keels, reflexed standards, paired dark-brown spots at the bottom of the standard blades and dorsiventrally flattened, equally 5-lobed calyces. (B) Laterally flattened and somewhat inflated fruit. (C) Succulent stem. (D) Leaf-opposed inflorescences and pinnately trifoliolate leaves. (E) Habit; biennial or short-lived perennial succulent shrublet. Photos: W. Swanepoel.

*Oberholzeria* is taxonomically isolated and its phylogenetic placement within the Crotalarieae is not supported by the molecular results. An analysis of the plastid *mat*K gene showed strong support (100% BS, 1.0 PP) for a sister group relationship to the Genisteae. The nuclear ITS analysis indicated that *Oberholzeria* is the sister group to both tribes, although this placement is not as strongly supported (76% BS, 1.0 PP). Due to the conflicting placements between the plastid and nuclear results, we have decided to compare the morphology of *Oberholzeria* with taxa from both tribes. The general morphology (Figs. 5 and 6) suggests an ancestral relationship based on shared characters with Genisteae and Crotalarieae. It is therefore necessary to compare the morphology of the new genus with the early-divergent taxa from both tribes.

The relationship between *Dichilus* DC. and *Melolobium* Eckl. & Zeyh. (Genisteae) is not resolved by our analyses, with alternating placements as early divergent taxa in this tribe. Consequently, we compared the new genus to both genera. *Oberholzeria* shares the following characters with *Dichilus* and *Melolobium*: Calyx shorter than the corolla; standard strongly reflexed (not strongly reflexed in *Melolobium*); anthers dimorphic; and fruits flat or slightly inflated [3, 6, 15, 28, 29].

*Dichilus* and *Melolobium* were previously included in the Crotalarieae [3, 4, 6] because of the structure of the staminal sheath, where all stamens are fused into a tube that is open along the upper side. In subsequent research done on the Crotalarieae and Genisteae, it was found that stamen fusion is taxonomically less important than previously considered and these two genera were moved to Genisteae [15]. In *Oberholzeria* the stamens are fused into a closed tube, which is a character state commonly found in other genera of the Genisteae [3, 9] but not in *Dichilus* or *Melolobium*. *Oberholzeria* also differs from these two genera in having a succulent, glabrous and unarmed habit (herbaceous and hairy in *Dichilus* and *Melolobium*, armed in the latter); pinnately trifoliolate leaves (digitately trifoliolate in *Dichilus* and *Melolobium*); five equally lobed calyces with dorsiventrally flattened lobes (bi-lobed and campanulate in *Dichilus* and *Melolobium*); callosities absent from the base of the standard lamina (callosities present in *Dichilus* only); wing petals longer than the keel and without spurs (shorter than the keel and with spurs in *Dichilus* and equal or longer than the keel but without spurs in *Melolobium*); rostrate keel apex (rounded or with blunt apices in *Dichilus* and *Melolobium*); and obovate-clavate, pendent and one-seeded fruit (narrowly oblong-linear to ovate, usually pointing upwards and more than one-seeded in *Dichilus* and *Melolobium*, rarely one-seeded in the latter).

Although *Crotalaria* L. is not the earliest-diverging taxon in the Crotalarieae, *Oberholzeria* superficially resembles this genus the most in general appearance (equally-lobed calyx, rostrate keel, 5 + 5 anther arrangement and oblong-clavate fruit [8, 23]). When compared with two additional, early-diverging taxa from this clade, *Euchlora* Eckl. & Zeyh. and *Bolusia* Benth., *Oberholzeria* shares the following characters: Equally lobed calyx (with the exception of certain taxa in *Crotalaria*); rostrate keel; 5 + 5 anther arrangement; and pendent fruit.

*Oberholzeria* also differs from *Euchlora*, *Bolusia* and *Crotalaria* in its succulent habit (herbaceous but non-succulent in *Euchlora*, *Bolusia* and *Crotalaria*); pinnately compound leaves (simple or digitately compound in *Euchlora*, *Bolusia* and *Crotalaria*); calyx with lobes dorsiventrally flattened, distally widely spreading and appearing stellate (campanulate in *Euchlora*, *Bolusia* and *Crotalaria*); standard without callosities (also absent in *Euchlora*, single callosity in *Bolusia* and paired callosities in *Crotalaria*); keel beak flat (flat in *Euchlora*, coiled in *Bolusia* and flat or sometimes twisted in *Crotalaria*); filaments of stamens fused into a closed tube (stamens fused into a tube that is open along the upper margin in *Euchlora*, *Bolusia* and *Crotalaria*); style glabrous (glabrous in *Euchlora* and *Bolusia*, hairy in almost all species of *Crotalaria*), fruit slightly inflated (strongly inflated in *Euchlora*, *Bolusia* and *Crotalaria* with only a few exceptions in the latter with flattened fruit).

*Oberholzeria etendekaensis* grows in localized patches of stony soil and scree (see under "Distribution, habitat and ecology" further on) within a semi-desert region of which the vegetation is fire intolerant, comprised of sparsely scattered perennial shrublets, shrubs and trees. Ephemerals and succulents are also present. The specific habitat of our new species is even more sparsely vegetated than the prevailing matrix vegetation, and temperatures here are suspected to be higher due to the rocky terrain. This habitat is best assigned as a local, environmentally harsher ecological anomaly within the succulent biome, one of four biomes recognized by Schrire and co-workers [30] as generalised areas of endemism predictive of legume distribution. The succulent habit of this new legume is remarkable in that succulence is rare in legumes. However, the legume family is particularly diverse in areas of the world where other succulent plant species are abundant and diverse. Following Schrire and co-workers’ study of *Indigofera* [31], an argument could be made that lineages endemic to patches of the succulent biome are expected to be evolutionary persistent because of the highly dispersal-limited nature of this biome and the absence of corridors to more suitable biomes. Thus, long branch lengths and phylogenetically isolated positions often characterise succulent biome endemics. This is indeed the case for *O*. *etendekaensis*, clearly a taxonomically isolated relictual species confined to a specialized habitat.

A summary of the most prominent diagnostic characters for these taxa is presented in Table 2; information on biomes in this table follows Schrire and co-workers [30]. It is clear from the morphology that some characters of *Oberholzeria* fit better with the Crotalarieae than with the Genisteae but there is stronger molecular support for its placement with the Genisteae than the Crotalarieae. This incongruent pattern is also reflected in the different placements in the phylogenies based on analyses of plastid and nuclear sequence data. It is therefore difficult to include this new genus with certainty in either tribe, although the *mat*K phylogeny provides much stronger support for a sister relationship to the Genisteae. We recommend the inclusion of *Oberholzeria* in the Genisteae but further studies, with more extensive sampling, are required to further clarify this relationship. More taxonomic evidence might even suggest it belongs to a new monogeneric tribe at the base of the Crotalarieae-Genisteae.

**Table 2:** Prominent differences between *Oberholzeria* and the early divergent genera from both the tribes Genisteae (*Dichilus* and *Melolobium*) and Crotalarieae (*Euchlora*, *Bolusia* and *Crotalaria*).

Character	Character state	Genus					
		*Oberholzeria*	*Dichilus*	*Melolobium*	*Euchlora*	*Bolusia*	*Crotalaria*
**Habit**	**- Succulent**	-	+	+	+	+	+
	**+ Herbaceous (but non-succulent)**						
**Leaves**	**- Pinnately compound**	-	+	+	+/++	+	+/(++)
	**+ Digitately compound**						
	**++ Simple**						
**Calyx (symmetry)**	**- Equally five-lobed**	-	+	+	-	-	-/(+)
	**+ Bilabiate**						
**Calyx (shape)**	**- Stellate (lobes widely spreading)**	-	+	+	+	+	+
	**+ Campanulate**						
**Callosities (on standard lamina)**	**- Absent**	-	+	-	-	+	+
	**+ Present**						
**Standard**	**- Strongly reflexed**	-	-	+	+	+	+
	**+ Not strongly reflexed**						
**Wing petals**	**- Longer than keel**	-	++	-/+	-	++	-/+/++
	**+ Equal to keel**						
	**++ Shorter than keel**						
**Staminal tube**	**- Fused; without a slit**	-	+	+	+	+	+
	**+ Fused; open along upper margin**						
**Anther configuration**	**- 5 + 5**	-	+	+	++	-	-
	**+ 4 + 1 + 5**						
	**++ 4 + 6**						
**Style surface**	**- Glabrous**	-	-	-	-	-	+
	**+ Hairy**						
**Fruit shape**	**- Obovate-clavate**	-	+	+	-/+	-/+	-/(+)/++
	**+ Oblong to linear-oblong**						
	**++ Round (in two dimensions)**						
**Fruit inflation**	**- Compressed or slightly inflated**	-	-	-	+	+	(-)/+
	**+ Highly inflated**						
**Fruit orientation**	**- Pendent**	-	+	+	-	-	-
	**+ Pointing to the sides/upwards**						
**Habitat**	**- Succulent Biome**	-	(-)/+	-/+/+++	-/+++	-/+	(-)/+/(++)/ (+++)
	**+ Grass Biome**						
	**++ Rainforest Biome**						
	**+++ Temperate Biome**						

Biomes referred to under habitat follow Schrire and co-workers [30].

## Taxonomic Treatment

***Oberholzeria*** Swanepoel, M.M.le Roux, M.F.Wojc. & A.E.van Wyk, *gen*. *nov*. [urn:lsid:ipni.org: names: 77145129–1] (Figs. 1, 5 and 6). Type:—*Oberholzeria etendekaensis* Swanepoel, M.M.le Roux, M.F.Wojc. & A.E.van Wyk, here designated.

Differs from *Dichilus* and *Melolobium* in the following suite of characters: Plants invariably succulent, glaucous, glabrous and unarmed; leaves fleshy and pinnately compound; calyx equally five-lobed, lobes reflexed and dorsiventrally flattened; standard lacking callosities at the base of the lamina; wings longer than keel; keel apex rostrate; filaments of stamens fused into a closed tube; and fruit obovate-clavate and one-seeded. In *Dichilus* and *Melolobium* the plants are herbaceous and hairy but armed in *Melolobium*; leaves not fleshy and digitately compound, calyx bilabiate, lobes not strongly reflexed, campanulate; standard with callosities at the base of the lamina in *Dichilus*, callosities absent in *Melolobium*; wings shorter than keel in *Dichilus*, wings equal to longer than the keel in *Melolobium*; keel apex obtuse; filaments of stamens fused into a tube with a slit in the sheath on the upper side; and fruit oblong to linear-oblong and more than one-seeded.

*Oberholzeria* also shares morphological characters with genera in the tribe Crotalarieae but differs from the early-divergent members of the clade *Euchlora*, *Bolusia* and *Crotalaria* in the following characters: Plants succulent; leaves pinnately compound; calyx dorsiventrally flattened, equally five-lobed with lobes distally widely spreading (calyx appearing stellate), standard without callosities; keel beak flat; filaments of stamens fused into a closed tube; style glabrous, fruit slightly inflated. In *Euchlora*, *Bolusia* and *Crotalaria* the plants are herbaceous; leaves simple to digitately compound; calyx campanulate and equally five-lobed but sometimes bilabiate in *Crotalaria*; standard without callosities in *Euchlora*, a single callosity present in *Bolusia* and paired callosities in *Crotalaria*; keel beak flat in *Euchlora*, coiled in *Bolusia* and flat or twisted in *Crotalaria*; filaments of stamens fused into a tube that is open along the upper margin; style glabrous in *Euchlora* and *Bolusia* but rarely glabrous in *Crotalaria*; fruit markedly inflated with only a few exceptions in *Crotalaria*.

Erect, single-stemmed, biennial or short-lived perennial succulent, up to 1 m tall, 1.2 m diam., glabrous. Stem and branches fleshy, yellow-green, with woody remains, lower branches deciduous, leaving prominent crescent-shaped scars, stem up to 0.4 m tall before branching. Stipules paired, linear-lanceolate or linear-triangular, 2.0–2.4 × 0.3–0.4 mm, fleshy, deciduous, stipels absent. Leaves spirally arranged, pinnately trifoliolate, leaflets often patent and erect; lamina ovate, rarely suborbicular, fleshy, glabrous, green or glaucous with a white bloom, 10–25 × 9–22 mm, lateral leaflets slightly smaller than terminal leaflet, margin entire, venation somewhat cladodromous, 3–7 lateral veins on each side, midrib prominent abaxially, lateral veins less so; base subcordate or truncate, apex obtuse or retuse, mucronulate abaxially, petiole 7–30 mm long, rachis 4–17 mm long, petiolules 1–3 mm long, petiolule of lateral leaflets 1–3 mm long, petiole and petiolules fleshy, petiolule of terminal leaflet inflexed proximally; strong pea-like scent when crushed. Inflorescences leaf-opposed due to sympodial growth, terminally disposed on young branches and branchlets, racemose, with 35–65 flowers, petals white with yellow-green venation; rachis 25–50 mm long, peduncle 9–17 mm long; flowers spirally arranged, each subtended by a lanceolate, fleshy, caducous bract, 2.6–3.0 × 0.7–0.9 mm, glabrous or adaxially with few tortuous hairs; bracteoles absent; pedicels 10–12 mm long and 0.5 mm wide. Calyx dorsiventrally flattened, 3.5–4.4 × 3.7–4.1 × 1.9–2.0 mm with five triangular lobes that are longer than the tube, lobes 2.3–3.2 × 1.5–1.7 mm, carinal lobe longest, sinuses equal, lobes distally widely spreading (calyx appearing stellate), glabrous or with few tortuous hairs adaxially. Standard narrowly obovate, retuse (folded medially, appearing oblanceolate in-situ), 6.2–7.5 × 2.9–3.2 mm, reflexed, lamina folded medially towards the apex, basal part fleshy, claw broad, indistinct and cucullate, lamina white with yellow in the central part and paired large dark-brown spots towards the basal margins. Wings broadly falcate, lamina 7.1–7.5 × 3.1–3.5 mm, longer than keel, auriculate at base with five columns of 3–12 crescent-shaped minute intercostal pockets; claw short but distinct, ± 0.6 mm long. Keel rostrate, lamina 5.7–5.9 × 2.6 mm, apex yellow to brown; claw short but distinct, 0.7–1.0 mm long. Stamens monadelphous, fused into a sheath that is closed above, anthers dimorphic, five long basifixed anthers, narrowly ovate, 0.8–1.4 × 0.5–0.6 mm, filaments up to 2.5 mm long, alternating with five short dorsifixed anthers, oblong, 0.4–0.6 × 0.3 mm, filaments up to 4.2 mm long. Ovary shortly stipitate, ventricose, ± 2.2 × 0.8 mm, with two ovules, style terete, tapering towards the stigma, slightly curved upwards, ± 2.2 mm long, glabrous; stigma terminal, penicillate, small, 0.15 mm diam. Fruit obovate-clavate, ± 23 × 14 × 6 mm, laterally flattened, slightly inflated, pendent, single seeded, green, khaki-coloured when dry, valves thin and papery, indehiscent with persistent calyx. Seeds asymmetrically obovate, laterally compressed, ± 8 × 7 × 3 mm, faintly verrucose, khaki or khaki-green, hilum not fleshy, cream-coloured, funicles ± 1.3 mm long.

***Oberholzeria etendekaensis*** Swanepoel, M.M.le Roux, M.F.Wojc. & A.E.van Wyk, sp. nov. [urn:lsid:ipni.org: names: 77145130–1] (Figs. 2–4). Type:—NAMIBIA. Kunene Region: Etendeka Mountains, 32 km NNW of Puros, 1812 (–DB), 850 m, 3 May 2012, *Swanepoel 316* (HOLOTYPE: WIND; ISOTYPE: PRE).

Description: Same as for the genus. Figs. 1, 5 and 6.

### Distribution, habitat and ecology

*Oberholzeria etendekaensis* is known from a single population (comprising two small subpopulations) in the Kaokoveld Centre of Endemism, a biogeographical region rich in restricted-range plants and animals [32], in north-western Namibia (Fig. 2). Its only known locality is from the Great Escarpment, in the Etendeka Mountains, on the watershed between the Khumib and Hoarusib Rivers, ± 50 km from the Atlantic coast at elevations ranging from 850–950 m. Average annual rainfall is around 100 mm and the substrate is derived from basalt of the Etendeka Group, Karoo Supergroup [33]. *Oberholzeria etendekaensis* is rare and has only been found in one location. It grows in two south-facing ravines (two subpopulations), approximately 500 m apart, in association with another succulent, *Euphorbia pergracilis* Meyer [34], also a restricted-range species. It is found on stony soil and scree in small colonies of usually less than ten plants each, in full sun (Fig. 1).

Flowering occurs in April and May; this follows the end of the main rainy season (late summer).

### Conservation status

Although rare and localised, *O*. *etendekaensis* does not appear to be threatened at present. No signs of browsing by livestock or game were noticed and plants seem to be healthy and occur in an area unpopulated or sparsely populated by humans.

### Etymology

Johanna Allettha Oberholzer [1965–], beloved and venerated wife of the first author, is commemorated in the genus name. Known as Hannelie, she proposed that this specific part of the Etendeka Mountains be explored, accompanied the expedition during which the new species was discovered and first saw and brought the plants to the attention of one of us (WS). The specific epithet refers to the Etendeka Mountains, the type locality of the new species.

## Supporting Information

S1 Table Collection details of voucher specimens used to generate new sequences during the current study. (DOCX)

S2 Table List of accessions used in the study of the ITS region. Included are the taxon name, respective GenBank number and the place of publication, or alternatively collector name, number and the herbarium where the voucher was deposited in the case of newly generated sequences.
(DOCX)

S3 Table List of accessions used in the study of the *mat*K region. Included are the taxon name, respective GenBank number and the place of publication, or alternatively the collector name, number and herbarium where the voucher was deposited in the case of newly generated sequences.
(DOCX)
