# Supplementary material for: Oberholzeria (Fabaceae subfam. Faboideae), a New Monotypic Legume Genus from Namibia
Source: PLoS One. 2015 Mar 27;10(3):e0122080. doi: 10.1371/journal.pone.0122080 (PMC4376691; doi:10.1371/journal.pone.0122080)
Supplement: S1 Table — (DOCX) [file pone.0122080.s001.docx]

**Table S1. Collection details of voucher specimens used to generate new sequences during the current study.** (DOC)

| **Taxon** | **Collector name** | **Collector number** | **Herbarium** | **Country** | **State/province** | **General locality** | **Year** |
| --- | --- | --- | --- | --- | --- | --- | --- |
| *Argyrolobium transvaalense* Schinz | A.R. Gotze | 298 | PRE | South Africa | Limpopo | Sekhukhuneland, Farm Zwartkoppies | 11/02/2010 |
| *Argyrolobium tuberosum* Eckl. & Zeyh. | S.P. Bester | 10865 | PRE | South Africa | Mpumalanga | Farm Haverklip 265 IR. Road crossing over Wilge River, ± 2 km SE of Delmas Colliery and 23 km SE of Delmas | 17/12/2011 |
| *Argyrolobium wilmsii* Harms | S. Krynauw | 584 | PRE | South Africa | Limpopo | The Downs 34 KT; Ca. 3 km from Makwens homestead | 22/05/1985 |
| *Leobordea eriantha* (Benth.) B.-E.van Wyk & Boatwr. | D. Cardoso et al. | 3273 | HUEFS | South Africa | Mpumalanga | Long Tom Pass, Regional Road R37, entre Lydenburg e Sabie | 13/01/2013 |
| *Leobordea hirsuta* (Schinz) B.-E.van Wyk & Boatwr. | S.P. Bester | 11922 | PRE | South Africa | Gauteng | Pretoria National Botanical Gardens, NE side of large piece of natural grassland. LATLONG: 25°44'17" S, 28°16'44" E | 07/04/2014 |
| *Melolobium calycinum* Benth. | A.E. van Wyk | 13662 | PRU | South Africa | North West | Leeuwfontein, ca. 10 km W of Wolmaransstad | 04/04/2014 |
| *Melolobium calycinum* Benth. | A.E. van Wyk | 13663 | PRU | South Africa | North West | Leeuwfontein, ca. 10 km W of Wolmaransstad | 04/04/2014 |
| *Melolobium calycinum* Benth. | A.E. van Wyk | 13664 | PRU | South Africa | North West | Leeuwfontein, ca. 10 km W of Wolmaransstad | 04/04/2014 |
| *Oberholzeria etendekaensis* Swanepoel, M.M.le Roux & A.E.van Wyk 1 | W. Swanepoel | 316-1 | WIND | Namibia | Kunene | Etendeka Mountains, 32 km NNW of Puros | 03/05/2012 |
| *Oberholzeria etendekaensis* Swanepoel, M.M.le Roux & A.E.van Wyk 2 | W. Swanepoel | 316-2 | WIND | Namibia | Kunene | Etendeka Mountains, 32 km NNW of Puros | 23/03/2013 |
| Pearsonia aristata (Schinz) Dümmer | D. Cardoso et al. | 3272 | HUEFS | South Africa | Mpumalanga | Nelspruit, Havelock Road | 13/13/2013 |
| *Pearsonia obovata* (Schinz) Polhill | D. Cardoso et al. | 3274 | HUEFS | South Africa | Mpumalanga | Long Tom Pass, Regional Road R37, entre Lydenburg e Sabie | 13/13/2013 |
| *Pearsonia sessilifolia* (Harv.) Dümmer | D. Cardoso et al. | 3271 | HUEFS | South Africa | Mpumalanga | Nelspruit, Havelock Road | 13/13/2013 |
| *Sellocharis paradoxa* Taub. | R. Lüdtke & M.S. Pereira | 488 | ICN | Brazil | Rio Grande do Sul | Viamão, Parque Estadual de Itapuã | 22/12/2005 |
