# Supplementary material for: Oberholzeria (Fabaceae subfam. Faboideae), a New Monotypic Legume Genus from Namibia
Source: PLoS One. 2015 Mar 27;10(3):e0122080. doi: 10.1371/journal.pone.0122080 (PMC4376691; doi:10.1371/journal.pone.0122080)
Supplement: S2 Table — Included are the taxon name, respective GenBank number and the place of publication, or alternatively collector name, number and the herbarium where the voucher was deposited in the case of newly generated sequences. (DOCX) [file pone.0122080.s002.docx]

| **Taxon** | **Genbank number - ITS region** | **Publication/voucher** |
| --- | --- | --- |
| *Adenocarpus viscosus* Webb & Berthel. | Z72300 and Z72301 | Käss (1995) |
| *Amphithalea biovulata* (H.Bolus) Granby | AM261219 | Boatwright et al. (2008a) |
| *Amphithalea speciosa* Schltr. | AM261235 | Boatwright et al. (2008a) |
| *Amphithalea tomentosa* (Thunb.) Granby | AM261430 | Boatwright et al. (2008a) |
| *Amphithalea muraltioides* (Benth.) A.L.Schutte | AM261230 | Boatwright et al. (2008a) |
| *Amphithalea vlokii* (A.L.Schutte & B.-E.van Wyk) A.L.Schutte | AM261435 | Boatwright et al. (2008a) |
| *Anagyris foetida* L. | AY091571 | Wang et al. (2006) |
| *Anarthrophyllum cumingii* F.Phil. | AY609186 and AY609196 | Ainouche and Misset s.n. |
| *Argyrocytisus battandieri* (Maire) Raynaud | Z95580 and Z95581 | Käss and Wink (1997) |
| *Argyrolobium lunare* Druce | AF287686 | Crisp et al. (2000) |
| *Argyrolobium tuberosum* Eckl. & Zeyh. | KP230719 | Bester 10865 (PRE) |
| *Argyrolobium harmsianum* Schltr. ex Harms | AF287685 | Crisp et al. (2000) |
| *Argyrolobium transvaalense* Schinz | KP230720 | Gotze 298 (PRE) |
| *Argyrolobium wilmsii* Harms | KP230721 | Krynauw 584 (PRE) |
| *Aspalathus macrantha* Harv. | EU347728 | Boatwright et al. (2008b) |
| *Aspalathus linearis* (Burm.f.) R.Dahlgren | EU347739 | Boatwright et al. (2008b) |
| *Aspalathus cordata* (L.) R.Dahlgren | AF287681 | Crisp et al. (2000) |
| *Baptisia australis* (L.) R. Br. | AY091572 | Wang et al. (2006) |
| *Baptisia tinctoria* (L.) Vent. | Z72314 and Z72315 | Käss (1995) |
| *Bolusanthus speciosus* Harms | AM262451 | Motsi (2004) |
| *Bolusia amboensis* Harms | JQ067344 | Le Roux et al. (2013) |
| *Brongniartia alamosana* Rydb. | AF467022 | Hu et al. (2002) |
| *Cadia pedicellata* Baker | AM261738 | Boatwright et al. (2008a) |
| *Cadia commersoniana* Baill. | AM261737 | Boatwright et al. (2008a) |
| *Cadia purpurea* (G. Piccioli) Aiton | AM261740 | Boatwright et al. (2008a) |
| *Calicotome villosa* (Poir.) Link | Z72252 and Z72253 | Käss (1995) |
| *Calpurnia sericea* Harv. | AM268374 and AM268375 | Boatwright et al. (2008a) |
| *Calpurnia aurea* Benth. | AJ409913 | Van der Bank et al. (2002) |
| *Calpurnia glabrata* Brummitt | AM177372 | Boatwright et al. (2008a) |
| *Crotalaria laburnifolia* L. | JQ067130 | Le Roux et al. (2013) |
| *Crotalaria lanceolata* E.Mey. | JQ067145 | Le Roux et al. (2013) |
| *Crotalaria lotoides* Benth. | JQ067299 | Le Roux et al. (2013) |
| *Crotalaria aurea* Dinter ex Baker f. | JQ067157 | Le Roux et al. (2013) |
| *Crotalaria juncea* L. | JQ067144 | Le Roux et al. (2013) |
| *Crotalaria novae-hollandiae* DC. | JQ067289 | Le Roux et al. (2013) |
| *Cyclolobium nutans* Rizzini & Heringer | AF467041 | Hu et al. (2002) |
| *Cyclopia genistoides* (L.) Vent. | AM050819 | Boatwright et al. (2008a) |
| *Cyclopia subternata* Vogel | AM050821 | Boatwright et al. (2008a) |
| *Cytisophyllum sessilifolium* O.Lang | Z72254 and Z72255 | Käss (1995) |
| *Dichilus strictus* E.Mey. | AF287684 | Crisp et al. (2000) |
| *Dichilus lebeckioides* DC. | EU347894 | Boatwright et al. (2008a) |
| *Dicraeopetalum mahafaliense* (M.Peltier) Yakovlev | EF457716 | Edwards and Hawkins (2007) |
| *Diplotropis martiusii* Benth. | AY553711 | Beck, Henner, and Jo. Cardosa 166 (US) |
| *Echinospartum boissieri* (Spach) Rothm. | AY609188 and AY609193 | MAF 148150 (Univ Complutense, Madrid) |
| *Erinacea anthyllis* Link | Z72256 and Z72257 | Käss (1995) |
| *Euchlora hirsuta* Druce | EU347881 | Boatwright et al. (2008b) |
| *Ezoloba macrocarpa* (Eckl. & Zeyh.) B.-E.van Wyk & Boatwr. | FM875935 and FM875936 | Boatwright et al. (2011) |
| *Genista teretifolia* Willk. | AY263668 | Pardo et al. (2004) |
| *Genista tournefortii* Spach | AY263669 | Pardo et al. (2004) |
| *Hesperolaburnum platycarpum* (Maire) Maire | AY263678 | Pardo et al. (2004) |
| *Hovea elliptica* (Sm.) DC. | AF287640 | Crisp et al. (2000) |
| *Laburnum anagyroides* Medik. | AY263679 | Pardo et al. (2004) |
| *Lebeckia wrightii* Bolus | AM262447 | Motsi (2004) |
| *Lebeckia sessilifolia* (Eckl. & Zeyh.) Benth. | AF287678 | Crisp et al. (2000) |
| *Lebeckia sepiaria* (L.) Thunb. | EU347853 | Boatwright et al. (2008b) |
| *Leobordea benthamiana* (Dümmer) B.-E.van Wyk & Boatwr. | EU347771 | Boatwright et al. (2008b) |
| *Leobordea eriantha* (Benth.) B.-E.van Wyk & Boatwr. | EU347784 | Boatwright et al. (2008b) |
| *Leobordea hirsuta* (Schinz) B.-E.van Wyk & Boatwr. | EU347881 | Boatwright et al. (2008b) |
| *Liparia vestita* Thunb. | AM261492 | Boatwright et al. (2008a) |
| *Liparia calycina* (L.Bolus) A.L.Schutte | AM261481 | Boatwright et al. (2008a) |
| *Liparia congesta* A.L.Schutte | AM261484 | Boatwright et al. (2008a) |
| *Listia marlothii* (Engl.) B.-E.van Wyk & Boatwr. | EU347825 | Boatwright et al. (2008b) |
| *Listia heterophylla* E.Mey. | EU347826 | Boatwright et al. (2008b) |
| *Lotononis laxa* Eckl. & Zeyh. | AF287677 | Crisp et al. (2000) |
| *Lotononis alpina* (Eckl. & Zeyh.) B.-E.van Wyk | AM262446 | Boatwright et al. (2008a) |
| *Lupinus polyphyllus* Lindl. | AF007496 | Ainouche and Bayer (1999) |
| *Lupinus arcticus* S.Watson | AF007495 | Ainouche and Bayer (1999) |
| *Maackia amurensis* Rupr. | Z72336 and Z72352 | Käss (1995) |
| *Melolobium candicans* Eckl. & Zeyh. | AM050833 | Moteetee (2003) |
| *Melolobium adenode*s Eckl. & Zeyh. | AM050832 | Moteetee (2003) |
| *Melolobium exudans* Harv. | KP230725 | Manning and Manning 2857 (K) |
| *Melolobium calycinum* Benth. | KP230723 | Van Wyk 13662 (PRU) |
| *Melolobium calycinum* Benth. | KP230724 | Van Wyk 13663 (PRU) |
| *Oberholzeria etendekaensis* Swanepoel, M.M.le Roux , M.F.Wojc. & A.E.van Wyk | KP247506 | Swanepoel 316 (WIND) |
| *Ormosia amazonica* Ducke | EF457724 | Edwards and Hawkins (2007) |
| *Pearsonia grandifolia* (Bolus) Polhill | AM262450 | Boatwright et al. (2008a) |
| *Pearsonia sessilifolia* Dümmer | AJ287675 | Crisp et al. (2000) |
| *Petteria ramentacea* C.Presl | Z72232 and Z72233 | Käss (1995) |
| *Pickeringia montana* Torr. & A. Gray | AY091568 | Wang et al. (2006) |
| *Piptanthus tomentosus* Franch. | AY091570 | Wang et al. (2006) |
| *Podalyria orbicularis* E.Mey. | AM261675 | Boatwright et al. (2008a) |
| *Podalyria argentea* Salisb. | AM261493 | Boatwright et al. (2008a) |
| *Podalyria myrtillifolia* Willd. | AJ409901 | Van der Bank et al. (2002) |
| *Poecilanthe falcata* (Vell.) Heringer | AF467492 | Hu et al. (2002) |
| *Polhillia pallens* C.H.Stirt. | EF457695 | Edwards and Hawkins (2007) |
| *Polhillia obsoleta* (Harv.) B.-E.van Wyk | KP230726 | Manning 2847 (K) |
| *Rafnia globosa* G.J.Campb. & B.-E.van Wyk | EU347743 | Boatwright et al. (2008b) |
| *Rafnia racemosa* Eckl. & Zeyh. | EU347741 | Boatwright et al. (2008b) |
| *Retama monosperma* (L.) Boiss. | AY263681 | Pardo et al. (2004) |
| *Retama sphaerocarpa* (L.) Boiss. | AY263682 | Pardo et al. (2004) |
| *Sophora velutina* Lindl. | FN813569 | Boatwright and Van Wyk (2011) |
| *Sophora inhambanensis* Klotzsch | FN813570 | Boatwright and Van Wyk (2011) |
| *Spartium junceum* L. | AF351088 | Cubas et al. (2002) |
| *Ulex genistoides* Brot. subsp. *genistoides* | AF384340 and AF384341 | Ainouche et al. (2003) |
| *Stirtonanthus insignis* (Compton) B.-E. van Wyk & A.L. Schutte | AJ409906 | Van der Bank et al. (2002) |
| *Stirtonanthus taylorianus* (L. Bolus) B.-E. van Wyk & A.L. Schutte | AJ409907 | Van der Bank et al. (2002) |
| *Stirtonanthus chrysanthus* (Adamson) B.-E.van Wyk & A.L. Schutte | AM268386 and AM268387 | Boatwright et al. (2008a) |
| *Styphnolobium japonicum* (L.) Schott | AJ409920 | Van der Bank et al. (2002) |
| *Templetonia retusa* (Vent.) R. Br. | AF287636 | Crisp et al. (2000) |
| *Templetonia hookeri* (F. Muell.) Benth. | GQ250085 | Queiroz et al. (2010) |
| *Thermopsis montana* Torr. & A. Gray | AF384336 and AF384337 | Ainouche et al. (2003) |
| *Thermopsis divaricarpa* A. Nelson | AY091575 | Wang et al. (2006) |
| *Ulex parviflorus* Pourr. | AF007470 | Ainouche and Bayer s.n. |
| *Ulex densus* Welw. ex Webb | AF384356 | Ainouche et al. |
| *Virgilia divaricata* Adamson | AJ409910 | Van der Bank et al. (2002) |
| *Virgilia oroboides* (P.J.Bergius) T.M.Salter subsp. *oroboides* | AJ409912 | Van der Bank et al. (2002) |
| *Xiphotheca canescens* (Thunb.) A.L.Schutte & B.-E.van Wyk | AM268388 and AM268389 | Boatwright et al. (2008a) |
| *Xiphotheca phylicoides* A.L.Schutte & B.-E.van Wyk | AM261743 | Boatwright et al. (2008a) |
| *Xiphotheca lanceolata* Eckl. & Zeyh. | AM261742 | Boatwright et al. (2008a) |
