# Supplementary material for: Oberholzeria (Fabaceae subfam. Faboideae), a New Monotypic Legume Genus from Namibia
Source: PLoS One. 2015 Mar 27;10(3):e0122080. doi: 10.1371/journal.pone.0122080 (PMC4376691; doi:10.1371/journal.pone.0122080)
Supplement: S3 Table — Included are the taxon name, respective GenBank number and the place of publication, or alternatively the collector name, number and herbarium where the voucher was deposited in the case of newly generated sequences. (DOCX) [file pone.0122080.s003.docx]

| **Taxon** | **Genbank number - *mat*K region** | **Publication/voucher** |
| --- | --- | --- |
| *Acosmium subelegans* (Mohlenbr.) Yakovlev | JX124410 | J.E. Meireles 489 (RB) |
| *Adenocarpus complicatus* J.Gay ex Gren. & Godr. | JQ858229 | Cunha et al. (2012) |
| *Ammodendron bifolium* (Pall.) Yakovlev | AY386957 | A. Whittemore s.n. (MONT) |
| *Ammopiptanthus mongolicus* (Maxim.) S.H.Cheng | JQ820169 | Strain 09050101 |
| *Ammopiptanthus nanus* (Popov) S.H.Cheng | JQ820170 | Strain 200802 |
| *Anagyris foetida* L. | KP230736 | Duran 6932 (KNYA) |
| *Anarthrophyllum desideratum* Benth. | AY386923 | Wojciechowski et al. (2004) |
| *Argyrolobium tuberosum* Eckl. & Zeyh. | KP230727 | Bester 10865 (PRE) |
| *Argyrolobium velutinum* Eckl. & Zeyh. | JQ412199 | JWB 503 |
| *Aspalathus pinguis* Thunb. | JQ412203 | JWB 512 |
| *Baptisia australis* R.Br. | AY386900 | Wojciechowski et al. (2004) |
| *Bolusanthus speciosus* Harms | AF142685 | Hu et al. (2000) |
| *Bolusia amboensis* Harms | JQ040984 | Le Roux et al. (2012) |
| *Bowdichia virgilioides* Knuth | AY386937 | Wojciechowski et al. (2004) |
| *Cadia purpurea* (G. Piccioli) Aiton | JX295932 | Cardoso et al. (2012) |
| *Calpurnia aurea* Benth. | AY386951 | Wojciechowski et al. (2004) |
| *Calpurnia sericea* Harv. | JX518205 | Abbott 9196 |
| *Camoensia brevicalyx* Benth. | JX295946 | Cardoso et al. (2012) |
| *Camoensia scandens* (Welw.) J.B.Gillett | JX295919 | Cardoso et al. (2012) |
| *Crotalaria incana* L. | GQ246141 | Queiroz et al. (2010) |
| *Crotalaria juncea* L. | JQ619982 | Bala s.n. (ASU) |
| *Crotalaria pumila* Ortega | AY386867 | Wojciechowski et al. (2004) |
| *Crotalaria saltiana* Andrews | JQ619981 | Chuang 4723 (ASU) |
| *Cyclopia genistoides* Sieber ex C.Presl | JX518243 | JWB 022 |
| *Cytisus hirsutus* L. | HE967392 | Bruni et al. (2012) |
| *Cytisus scoparius* (L.) Link | AY386902 | Wojciechowski et al. (2004) |
| *Dichilus lebeckioides* DC. | GQ246143 | Queiroz et al. (2010) |
| *Dicraeopetalum stipulare* Harms | GQ246142 | Queiroz et al. (2010) |
| *Diplotropis brasiliensis* (Tul.) Benth. | AY386939 | Wojciechowski et al. (2004) |
| *Diplotropis martiusii* Benth. | AY386938 | Wojciechowski et al. (2004) |
| *Euchlora hirsuta* Druce | JQ041113 | Le Roux et al. (2012) |
| *Genista anglica* L. | JN894663 | NMW 4174 |
| *Genista monspessulana* (L.) L.A.S.Johnson | AY386862 | Wojciechowski et al. (2004) |
| *Laburnum anagyroides* Medik. | HE967423 | Bruni et al. (2012) |
| *Lebeckia sericea* Thunb. | GQ246144 | Queiroz et al. (2010) |
| *Leobordea eriantha* (Benth.) B.-E.van Wyk & Boatwr. | KP230706 | Cardoso et al. 3273 (HUEFS) |
| *Leobordea hirsuta* (Schinz) B.-E.van Wyk & Boatwr. | KP230728 | S. P. Bester 11922 (PRE) |
| *Leptolobium dasycarpum* Vogel | JX124408 | Cardoso et al. (2012) |
| *Liparia myrtifolia* Thunb. | JX517632 | JWB 039 |
| *Liparia rafnioides* A.L.Schutte | JX517668 | JWB 033 |
| *Lupinus argenteus* Pursh | AY386956 | Wojciechowski et al. (2004) |
| *Lupinus brevicaulis* Griseb. | EU025879 | \| A. Tiehm 13819 (ASU) \| \| --- \| |
| *Lupinus cosentinii* Guss. | AY386943 | Wojciechowski et al. (2004) |
| *Lupinus flavoculatus* A.Heller | EU025898 | A. Tiehm 13537 (ASU) |
| *Lupinus huachucanus* M.E.Jones | EU025906 | M. Baker 13435 (ASU) |
| *Lupinus odoratus* A.Heller | EU025914 | Van Devender 86-111 (ARIZ) |
| *Lupinus tegeticulatus var. duranii* (Eastw.) Barneby | AY386910 | Wojciechowski et al. (2004) |
| *Maackia amurensis* Rupr. | AY386944 | Wojciechowski et al. (2004) |
| *Melolobium calycinum* Benth. 1 | KP230729 | Van Wyk 13662 (PRU) |
| *Melolobium calycinum* Benth. 2 | KP230730 | Van Wyk 13663 (PRU) |
| *Melolobium exudans* Harv. | KP230731 | Manning & Manning 2857 (K) |
| *Oberholzeria etendekaensis* Swanepoel, M.M.le Roux, M.F.Wojc. & A.E.van Wyk 1 | KP230732 | Swanepoel 316-1 (WIND) |
| *Oberholzeria etendekaensis* Swanepoel, M.M.le Roux, M.F.Wojc. & A.E.van Wyk 2 | KP230733 | Swanepoel 316-2 (WIND |
| *Pearsonia aristata* Dümmer | KP230707 | Cardoso et al. 3272 (HUEFS) |
| *Pearsonia obovata* (Schinz) Polhill | KP230708 | Cardoso et al. 3274 (HUEFS) |
| *Pearsonia sessilifolia* subsp. *marginata* (Schinz) Polhill | KP230709 | Cardoso et al. 3271 (HUEFS) |
| *Piptanthus nepalensis* Sweet | AY386924 | Wojciechowski et al. (2004) |
| *Podalyria calyptrata* Willd. | JX518039 | MWC 16091 |
| *Podalyria myrtillifolia* Willd. | JX517747 | AMM 5052 |
| *Polhillia obsoleta* (Harv.) B.-E.van Wyk | KP230734 | Manning 2847 (K) |
| *Rafnia angulata* Thunb. | JQ412281 | JWB 510 |
| *Sellocharis paradoxa* Taub. | KP230740 | Lüdtke & Pereira 488 (ICN) |
| *Sophora macrocarpa* Sm. | JQ619975 | Landrum 5855 (ASU) |
| *Sophora microphylla* Aiton | JQ619976 | Landrum 7622 (ASU) |
| *Sophora nuttalliana* B.L.Turner | AY386865 | Wojciechowski et al. (2004) |
| *Sophora stenophylla* A.Gray | JQ669580 | Gierisch 4997 (ASU) |
| *Spartium junceum* L. | AY386901 | Wojciechowski et al. (2004) |
| *Styphnolobium japonicum* (L.) Schott | AY386962 | Wojciechowski et al. (2004) |
| *Thermopsis alpina* Ledeb. | JQ669594 | Long et al., Sino-British Qinghai Exp. 497 (E) |
| *Thermopsis lanceolata* R.Br. | JQ669595 | Long et al., Sino-British Qinghai Exp. 526 (E) |
| *Thermopsis rhombifolia* (Nutt. ex Pursh) Richardson | AY386866 | Wojciechowski 807 (MONT) |
| *Ulex europaeus* L. | JQ669586 | Damrel 2304 (ASU) |
| *Ulex gallii* Planch. | JN895798 | NMW 4258 |
| *Virgilia divaricata* Adamson | JX517500 | OM 3169 |
